# P085 Polymyalgia rheumatica and late-onset axial spondyloarthritis: recognising diagnostic overlap

**DOI:** 10.1093/rap/rkag113.115

**Published:** 2026-11-03

**Authors:** Poh Chen Tan, Ashley Black, Jonathan McKnight, Maria Wray

**Affiliations:** Department of Rheumatology, Antrim Area Hospital, Northern Health and Social Care Trust, Antrim, United Kingdom; Department of Rheumatology, Antrim Area Hospital, Northern Health and Social Care Trust, Antrim, United Kingdom; Department of Rheumatology, Antrim Area Hospital, Northern Health and Social Care Trust, Antrim, United Kingdom; Department of Rheumatology, Antrim Area Hospital, Northern Health and Social Care Trust, Antrim, United Kingdom

## Abstract

**Introduction:**

Polymyalgia rheumatica (PMR) is an inflammatory disorder affecting adults over 50, causing pain and stiffness in the neck, shoulders, and hips. It shares some similar clinical features with axial spondyloarthritis (AxSpA), which typically begins before age 45 but can rarely present later, creating diagnostic challenges. Cases of PMR onset AxSpA have been reported. Misdiagnoses or delayed diagnosis may cause postponement in commencing appropriate disease modifying therapy and corticosteroid dependency. We describe a case of an elderly patient who initially presented with symptoms of PMR, with subsequent imaging demonstrating coexisting PMR and AxSpA, highlighting the importance of considering overlapping inflammatory conditions.

**Case description:**

A 70-year-old female presented with a 4-month history of neck, bilateral shoulder and hip girdle pain and stiffness associated with early morning stiffness, and no response to NSAIDs. She denied any cranial giant cell arteritis (GCA) symptoms or weight loss but admitted to night sweats and feeling feverish. She denied any peripheral joint symptoms. Initial blood test showed ESR 97 mm/hr and CRP 77 mg/L, CK minimally raised 484 U/L. She was diagnosed as PMR and given 15mg oral prednisolone, which she reported a 70% improvement, albeit her symptoms were still present, with normalisation of her inflammatory markers. However, symptoms of shoulder pain and stiffness recurred when she weaned her prednisolone dose to 10mg, along with rising inflammatory markers (between 20 and 30). Given her partial steroid response with constitutional symptoms, prednisolone was taper and FDG PETCT arranged.

Her FDG PETCT demonstrated inflammatory changes involving the cervical spine, costovertebral and sacroiliac joints, with additional shoulder and pelvic girdle uptake, suggesting AxSpA with possible concurrent PMR. Her MRI of spine and sacroiliac joints showed changes in SI joints and posterior aspects of vertebral bodies consistent with an inflammatory arthropathy. Her HLA-B27 was negative.

On further review, she recalled mild inflammatory back pain dating to her 20s following pregnancy. She had no psoriasis, inflammatory bowel disease, uveitis, family history of spondyloarthritis, and was an ex-smoker.

Physiotherapy was initiated and prednisolone was gradually tapered as per PMR schedule. She was worked up and commenced on adalimumab for her AxSpA.

Unfortunately she had a relapse of symptoms along with constitutional upset. A repeat FDG PETCT showed no evidence of large vessel vasculitis, but demonstrated persistent but mildly improved inflammatory arthropathy and enthesitis of the cervical and thoracic spine.

**Discussion:**

This case highlights a PMR onset presentation, with subsequent imaging showing both PMR and AxSpA, which is not widely recognised or described in clinical practice. AxSpA presenting in the elderly population is rare, but there have been a few case reports of patients diagnosed with what is now termed late onset AxSpA (lo-AxSpA), defined by symptom onset after 45 years of age. The prevalence of this group is increasing, possibly due to an ageing global population with longer lifespans, as well as improved diagnostic methods in medicine.

Lo-AxSpA can initially present as PMR, or it can present as RS3PE or pyrexia of unknown origin. Lo-AxSpA is phenotypically different from early onset AxSpA (eo-AxSpA). A study comparing the two groups showed that lo-AxSpA tends to affect women more than men, has a lower response rate to NSAIDs, less likely to have a family history of spondyloarthropathy, and a weaker association with HLA-B27. Lo-AxSpA patients also have more peripheral arthritis, but less acute anterior uveitis and inflammatory back pain than those with eo-AxSpA.

As PMR can mimic AxSpA and vice versa, it can be challenging to distinguish between the two conditions due to similar clinical presentations. As PMR has been historically a clinical diagnosis, unless there is suspicion of GCA symptoms, management involves moderate doses of prednisolone which is effective in treating this condition. Due to the dramatic response to prednisolone, little to no imaging is required. However, if patients are only reporting partial or suboptimal steroid responses, this should prompt re-evaluation of their symptoms and alternative diagnoses should be sought. MRI is invaluable in distinguishing AxSpA from PMR, if AxSpA is suspected. Treatment options of lo-AxSpA would be biological DMARD (bDMARD) with either an anti-TNF or IL-17 inhibitor as per BSR 2025 guidelines.

**Key learning points:**

• This case highlights the challenges of the ‘atypical PMR’ patient groups faced by clinicians, and the importance of having a low threshold to re-evaluate diagnoses if there is difficulty in tapering steroid doses, or responses to steroids are suboptimal.

• The case challenges our assumptions that AxSpA normally presents in younger males, but there have been reported cases of patients who have been diagnosed with late-onset AxSpA who initially presented as PMR, and once these patients have had their diagnoses revised, have responded well to bDMARD therapy.

• It also emphasises a potential diagnostic pitfall faced by clinicians, as the incorrect diagnosis can lead to unintended consequences such as delayed treatment, ongoing joint inflammation, steroid dependency, and the long-term side effects that comes with it.

